# Family economic hardship and adolescent mental health during the COVID-19 pandemic

**DOI:** 10.3389/fpubh.2022.904985

**Published:** 2022-09-06

**Authors:** Bomgyeol Kim, Do Hee Kim, Suk-Yong Jang, Jaeyong Shin, Sang Gyu Lee, Tae Hyun Kim

**Affiliations:** ^1^Department of Public Health, Graduate School, Yonsei University, Seoul, South Korea; ^2^Department of Healthcare Management, Graduate School of Public Health, Yonsei University, Seoul, South Korea; ^3^Department of Preventive Medicine, College of Medicine, Yonsei University, Seoul, South Korea

**Keywords:** adolescent, anxiety, COVID-19, depression, economic hardship, suicidal ideation

## Abstract

**Objective:**

This study examined whether pandemic related family economic hardships influenced adolescents' mental health during the COVID-19 pandemic in Korea.

**Methods:**

Data were collected from 54,948 adolescents who participated in the 2020 Korea Youth Risk Behavior Web-Based Survey. We performed a multiple logistic regression analysis to examine the association between family economic hardship and mental health (anxiety, depressive symptoms, and suicidal ideation).

**Results:**

Among the adolescents, 39.7, 24.7, and 5.9% reported slight, moderate, and severe economic hardship, respectively. COVID-19 related family economic hardship was significantly associated with higher odds of adolescents reporting anxiety, depressive symptoms, and suicidal ideation. This association was stronger among adolescents with low to middle family economic status.

**Conclusions:**

This study suggests that adolescents from more economically vulnerable families are likely to be at a higher risk for long-term mental health effects due to the financial consequences of the COVID-19 pandemic.

## Introduction

The COVID-19 (Coronavirus Disease 2019) pandemic has led to rapid, unprecedented changes in the lives of billions of adolescents (1). The first infection in Korea was detected on 20 January 2020, triggering a national response including school closures, home confinement, and social distancing rules (2, 3). Fluctuating school and family routines, isolation at home, stressed parents, and fear of the virus have impacted adolescents significantly. Indeed, increasing mental health problems in adolescents during the COVID-19 pandemic have been reported in Korea and other countries (4–7).

More importantly, adolescents' deteriorated mental health outcomes may reflect socioeconomic inequalities (specifically, the economic well-being of households) (8). During the prolonged COVID-19 lockdowns, many employed people faced heavily reduced workloads, temporary work suspensions such as furloughs, and even job loss (9). Economic activities were at a standstill, which can pose enormous challenges for the mental health of affected workers and their families (10, 11). Particularly, for adolescents, COVID-19-related family economic hardship is an independent and uncontrollable life event (12). Adolescents and their parents have different perceptions of the financial situation of the family. Moreover, the perceptions of adolescents seem to have a stronger association with adolescent mental health than the perceptions of parents (12, 13).

Many families have been significantly impacted economically by COVID-19, but poor families have been affected the most (14). However, it has been reported that all groups of households, from the poorest to the richest, have experienced declines in their incomes at a similar rate (15). Economically stable middle-income households are also at risk of becoming low-income households (15). Based on the results of various studies, the key remaining question is whether there is a difference in the relationship between COVID-19-related economic hardship and mental health depending on the household income level among adolescents.

Numerous family socioeconomic hardships have been linked to placing adolescents at risk for suffering poor mental health outcomes. The existing evidence points to gender, school grade, residential area, subjective academic performance, smoking status, alcohol use, and socioeconomic status being associated with mental health (16–20). In addition, children's social, emotional, and cognitive development can be affected by experiences of instability at home and school, emotional and sexual trauma (19), and domestic and community violence (18). These negative events may permanently affect a child's mental health development if they are frequent and severe. Studies related to the post-COVID-19 mental health of adolescents have found that isolation from peers, uncertainty regarding short-term and long-term prospects, and continuous states of fear, including the fear of being infected, pose a risk for developing psychopathology (5, 21, 22).

As the COVID-19 pandemic lingers, understanding the negative impact of the economic consequences on adolescents' mental health is imperative. However, there is a lack of empirical evidence on COVID-19-related family economic hardship and its influence on mental health during the pandemic. Particularly, this issue has never been studied in Korea. Thus, the present study aimed to identify the association between COVID-19-related family economic hardship from the perspective of adolescents' mental health in Korea. We hypothesized that family economic hardship due to the pandemic is associated with mental health problems among adolescents (H1). Further, we hypothesized that the association between COVID-19-related family economic hardship and adolescents' mental health differs according to the current subjective family economic status (H2).

## Methods

### Data and population

For this cross-sectional study, we collected data from the 2020 Korea Youth Risk Behavior Web-based Survey (KYRBWS), a national survey on various health-related behaviors of Korean adolescents. The KYRBWS is an anonymous, internet-based, self-administered, structured questionnaire (23). To obtain a representative sample, the KYRBWS designed a complex sample technique that included multiple stages, such as stratification, clustering, and multi-step sampling (23). Additionally, the KYRBWS weighted the students who participated in the survey so that they represent Korean adolescents, thereby, estimating the level of adolescents' health behavior. A representative sample of students from the 7–12^th^ grades, aged 13–18 years, was selected; the survey was developed for school-based samples according to city size, regional group, and school type among 16 major cities and provinces in Korea, and one sample class per grade level was randomly selected. Students were instructed to complete their questionnaire at their respective schools' computer labs during school hours, under the guidance of their teachers. Detailed information on the research design and methods of the KYRBWS is presented in a previous paper (23). From 2015, the ethics approval for the KYRBWS was waived by the Korea Centers for Disease Control and Prevention Institutional Review Board under the Bioethics & Safety Act and opened to the public for academic use.

The 2020 KYRBWS survey was conducted from 3 August 2020, to 13 November 2020. It was started about 6 months after the first confirmed COVID-19 case in Korea (20 January 2020) due to the impact of COVID-19, and the survey was conducted during the second COVID-19 wave (August to November 2020). The KYRBWS adheres to the Declaration of Helsinki and all participants provided informed consent (24). As the KYRBWS was conducted as an online survey, there were no non-response items in the original data; however, logical errors and outliers were treated as missing values. All surveyed 54,948 adolescent participants were included in the analysis without any missing values.

### Measurements

#### Dependent variables

The main dependent variable of this study was mental health, which included anxiety, depressive symptoms, and suicidal ideation. We screened for anxiety using the seven-item Generalized Anxiety Disorder scale (GAD-7), which is valid and reliable when applied to the general population (25, 26). The GAD-7 is rapidly becoming a gold-standard screening tool for general anxiety disorder measurements and has been shown to have acceptable specificity and sensitivity for detecting clinically significant anxiety in adolescents (27). The Cronbach's α for the GAD-7 was 0.90 in the current sample. Cronbach's alpha should be from 0.70 up to and including 0.90 to demonstrate adequate consistency in a scale (28). In this study, we used a GAD-7 cutoff score of ≥10, as this threshold provides a consistent reflection of anxiety levels (25). This study followed the questions and answers that were originally structured by the KYRBWS. We determined the presence of depressive symptoms using the question: “Have you felt sad or hopeless enough to stop your daily routine for 2 weeks in the past 12 months?” Participants responded to the question with either “yes” or “no.” Suicidal ideation and suicide attempts were assessed using the following two questions from the KYRBWS: “Have you seriously considered suicide in the past 12 months?” and “Have you attempted suicide in the past 12 months? The participants responded to both the questions with either “yes” or “no.”

#### Variable of interest

The variable of interest was COVID-19-related family economic hardship, which was assessed by the following question from the perspective of the children themselves: “Do you think the COVID-19 outbreak has caused your family economic status to be worsened?” The concept of COVID-19-related family economic hardship directly measured the nature and extent of deprivation that children were experiencing due to a lack of financial resources caused by COVID-19 and relative to their own needs (29). The variable of interest was categorized based on whether respondents answered these questions as “No,” “Slight,” “Moderate,” or “Severe.”

#### Control variables

The control variables used in this study were sex, school grade, residential area, co-residence with parents, subjective academic performance, subjective family economic status, subjective health status, smoking status, and alcohol use. Residential area was classified into “metropolitan,” “urban,” and “rural.” Co-residence with parents was classified into “yes” or “no.” Subjective academic performance and subjective family economic status were classified originally into: “high,” “upper-middle,” “middle,” “lower-middle,” and “low” and further classified into three categories in study, including: “high” (high and upper-middle), “middle,” and “low” (lower-middle and low). Subjective health status was measured with the question: “How would you rate your health in general?” The response options were “very good,” “good,” “normal,” “bad,” and “very bad” and further classified into three categories, including: “good” (very good and good), “normal,” and “bad” (bad and very bad). Smoking status and alcohol use were classified into “yes” and “no.”

### Statistical analysis

In this study, the statistical values were calculated using sample weights assigned to the participants. The KYRBWS constructed sample weights to represent the Korean adolescent population by accounting for the complex survey design and survey non-responses. We performed a Rao-Scott chi-square test to examine the bivariate associations between adolescents' mental health problems and selected covariates—the general characteristics of the study population. Further, we performed a multiple logistic regression analysis to analyze the association between COVID-19 related family economic hardship and mental health, after controlling for covariates. The results were reported using adjusted odds ratios (aORs) and confidence intervals (CIs). Model fitting was performed using the PROC SURVEYLOGISTIC procedure and application of cluster and strata. In addition, stratified analyses according to subjective family economic status were performed on the association between COVID-19-related family economic hardship and mental health using multiple logistic regression, adjusted by sex, grade, residential area, co-residence with parents, subjective academic performance, subjective health status, smoking status, and alcoholic drinking. All statistical analyses were performed using SAS v9.4 (SAS Institute Inc.; Cary, North Carolina). Statistical significance was set at *P* < 0.05.

## Results

The participants included in the analysis comprised 54,948 adolescents. Among them, 16,268 (29.6%) experienced no economic hardship related to COVID-19, 21,841 (39.7%) experienced slight economic hardship, 13,583 (24.7%) experienced moderate economic hardship, and 3,256 (5.9%) experienced severe economic hardship. Refer to Table 1 for an in-depth overview of the participants' characteristics.

**Table 1:** General characteristics of the Korean adolescents included in the analysis (*N* = 54,948).

		**Anxiety**					**Depressive symptoms**					**Suicidal ideation**				
**Variables**	**Total**		**Yes**		**No**		P^*^	**Yes**		**No**		P^*^	**Yes**		**No**		P^*^
	***N*** **(%)**		***N*** **(%)**		***N*** **(%)**			***N*** **(%)**		***N*** **(%)**			***N*** **(%)**		***N*** **(%)**		
**Total**	54,948	(100.0)	6,099	(11.1)	48,849	(88.9)		13,840	(25.2)	41,108	(74.8)		5,979	(10.9)	48,969	(89.1)	
**COVID-19-related** **family economic hardship**							<0.0001					<0.0001					<0.0001
No	16,268	(29.6)	1,419	(8.7)	14,849	(91.3)		3,435	(21.1)	12,833	(78.9)		1,394	(8.6)	14,874	(91.4)	
Slight	21,841	(39.7)	2,206	(10.1)	19,635	(89.9)		5,175	(23.7)	16,666	(76.3)		2,200	(10.1)	19,641	(89.9)	
Moderate	13,583	(24.7)	1,801	(13.3)	11,782	(86.7)		3,992	(29.4)	9,591	(70.6)		1,766	(13.0)	11,817	(87.0)	
Severe	3,256	(5.9)	673	(20.7)	2,583	(79.3)		1,238	(38.0)	2,018	(62.0)		619	(19.0)	2,637	(81.0)	
**Sex**							<0.0001					<0.0001					<0.0001
Male	28,353	(51.6)	2,191	(7.7)	26,162	(92.3)		5,633	(19.9)	22,720	(80.1)		2,254	(7.9)	26,099	(92.1)	
Female	26,595	(48.4)	3,908	(14.7)	22,687	(85.3)		8,207	(30.9)	18,388	(69.1)		3,725	(14.0)	22,870	(86.0)	
**School grade**							<0.0001					<0.0001					<0.0001
7	10,005	(18.2)	880	(8.8)	9,125	(91.2)		2,030	(20.3)	7,975	(79.7)		897	(9.0)	9,108	(91.0)	
8	9,564	(17.4)	1,010	(10.6)	8,554	(89.4)		2,281	(23.8)	7,283	(76.2)		1,063	(11.1)	8,501	(88.9)	
9	9,392	(17.1)	1,055	(11.2)	8,337	(88.8)		2,429	(25.9)	6,963	(74.1)		1,053	(11.2)	8,339	(88.8)	
10	8,907	(16.2)	922	(10.4)	7,985	(89.7)		2,244	(25.2)	6,663	(74.8)		926	(10.4)	7,981	(89.6)	
11	8,907	(16.2)	1,101	(12.4)	7,806	(87.6)		2,476	(27.8)	6,431	(72.2)		1,085	(12.2)	7,822	(87.8)	
12	8,173	(14.9)	1,131	(13.8)	7,042	(86.2)		2,380	(29.1)	5,793	(70.9)		955	(11.7)	7,218	(88.3)	
**Residential area**							<0.0001					<0.0001					0.0008
Metropolitan	23,621	(43.0)	2,414	(10.2)	21,207	(89.8)		5,709	(24.2)	17,912	(75.8)		2,440	(10.3)	21,181	(89.7)	
Urban	26,981	(49.1)	3,175	(11.8)	23,806	(88.2)		7,026	(26.0)	19,955	(74.0)		3,069	(11.4)	23,912	(88.6)	
Rural	4,346	(7.9)	510	(11.7)	3,836	(88.3)		1,105	(25.4)	3,241	(74.6)		470	(10.8)	3,876	(89.2)	
**Co-residence with parents**							0.0011					<0.0001					<0.0001
Yes	52,332	(95.2)	342	(13.1)	2,274	(86.9)		13,068	(25.0)	39,264	(75.0)		5,594	(10.7)	46,738	(89.3)	
No	2,616	(4.8)	5,757	(11.0)	46,575	(89.0)		772	(29.5)	1,844	(70.5)		385	(14.7)	2,231	(85.3)	
**Subjective academic performance**							<0.0001					<0.0001					<0.0001
High	20,146	(36.7)	1,974	(9.8)	18,172	(90.2)		4,406	(21.9)	15,740	(78.1)		1,942	(9.6)	18,204	(90.4)	
Middle	16,585	(30.2)	1,585	(9.6)	15,000	(90.4)		3,890	(23.5)	12,695	(76.5)		1,551	(9.4)	15,034	(90.6)	
Low	18,217	(33.2)	2,540	(13.9)	15,677	(86.1)		5,544	(30.4)	12,673	(69.6)		2,486	(13.6)	15,731	(86.4)	
**Subjective family economic status**							<0.0001					<0.0001					<0.0001
High	21,339	(38.8)	2,024	(9.5)	19,315	(90.5)		4,926	(23.1)	16,413	(76.9)		2,008	(9.4)	19,331	(90.6)	
Middle	26,397	(48.0)	2,743	(10.4)	23,654	(89.6)		6,385	(24.2)	20,012	(75.8)		2,639	(10.0)	23,758	(90.0)	
Low	7,212	(13.1)	1,332	(18.5)	5,880	(81.5)		2,529	(35.1)	4,683	(64.9)		1,332	(18.5)	5,880	(81.5)	
**Subjective health status**							<0.0001					<0.0001					
Good	38,444	(70.0)	2,842	(7.4)	35,602	(92.6)		8,065	(21.0)	30,379	(79.0)		3,049	(7.9)	35,395	(92.1)	<0.0001
Normal	12,342	(22.5)	1,955	(15.8)	10,387	(84.2)		3,892	(31.5)	8,450	(68.5)		1,799	(14.6)	10,543	(85.4)	
Bad	4,162	(7.6)	1,302	(31.3)	2,860	(68.7)		1,883	(45.2)	2,279	(54.8)		1,131	(27.2)	3,031	(72.8)	
**Smoking status**							<0.0001					<0.0001					<0.0001
No	52,478	(95.5)	5,663	(10.8)	46,815	(89.2)		12,741	(24.3)	39,737	(75.7)		5,432	(10.4)	47,046	(89.6)	
Yes	2,470	(4.5)	436	(17.7)	2,034	(82.4)		1,099	(44.5)	1,371	(55.5)		547	(22.1)	1,923	(77.9)	
**Alcoholic use**							<0.0001					<0.0001					<0.0001
No	49,056	(89.3)	5,096	(10.4)	43,960	(89.6)		11,505	(23.5)	37,551	(76.5)		4,809	(9.8)	44,247	(90.2)	
Yes	5,892	(10.7)	1,003	(17.0)	4,889	(83.0)		2,335	(39.6)	3,557	(60.4)		1,170	(19.9)	4,722	(80.1)	

* Chi-Square p value.

The greater the post-COVID-19 related family economic hardship reported by adolescents, the higher the mental health complaint scores. Anxiety was experienced by 1,419 (8.7%) adolescents who experienced no economic hardship, 2,206 (10.1%) who experienced slight economic hardship, 1,801 (13.3%) who experienced moderate economic hardship, and 673 (20.7%) who experienced severe economic hardship. Depressive symptoms were experienced by 3,435 (21.1%) adolescents who experienced no economic hardship, 5,175 (23.7%) who experienced a slight economic hardship, 3,992 (29.4%) who experienced moderate economic hardship, and 1,238 (38.0%) who experienced severe economic hardship. Suicidal ideation was found in 1,394 adolescents (8.6%) who experienced no economic hardship, 2,200 (10.1%) who experienced a slight economic hardship, 1,766 (13.0%) who experienced moderate economic hardship, and 619 (19.0%) who experienced severe economic hardship.

Table 2 reports the results of the multiple logistic regression analysis, which confirmed that COVID-19 related family economic hardship was significantly associated with increased odds of mental health problems. Adolescents who experienced a higher level of economic hardship than those without hardship showed a higher possibility of anxiety (experienced slight hardship aOR = 1.07, 95% CI: 0.98–1.17; experienced moderate hardship aOR = 1.29, 95% CI: 1.17–1.42; experienced severe hardship aOR = 2.09, 95% CI: 1.82–2.40). Similarly, among adolescents who experienced more hardship than those who did not, the likelihood of depressive symptoms increased (experienced slight hardship aOR = 1.10, 95% CI: 1.04–1.16; experienced moderate hardship aOR = 1.38, 95% CI: 1.29–1.48; experienced severe hardship aOR = 1.90, 95% CI: 1.72–2.09). Adolescents who experienced a higher level of economic hardship than those without any change in economic status exhibited a higher possibility of suicidal ideation (experienced slight hardship aOR = 1.09, 95% CI: 1.00–1.18; experienced moderate hardship aOR = 1.28, 95% CI: 1.17–1.40; experienced severe hardship aOR = 1.86, 95% CI: 1.64–2.11).

**Table 2:** Association between COVID-19-related family economic hardship and mental health (*N* = 54,948).

	**Anxiety**		**Depressive symptoms**		**Suicidal ideation**	
**Variables**	**aOR (95% CI)**	***P***	**aOR (95% CI)**	***P***	**aOR (95% CI)**	***P***
**COVID-19-related family economic hardship**						
No	1.00					
Slight	1.07 (0.98–1.17)	0.1312	1.10 (1.04–1.16)	0.0018	1.09 (1.00–1.18)	0.0432
Moderate	1.29 (1.17–1.42)	<0.0001	1.38 (1.29–1.48)	<0.0001	1.28 (1.17–1.40)	<0.0001
Severe	2.09 (1.82–2.40)	<0.0001	1.90 (1.72–2.09)	<0.0001	1.86 (1.64–2.11)	<0.0001

We adjusted the analyses for sex, grade, residential area, co-residence with parents, subjective academic performance, subjective family economic status, subjective health status, smoking status, alcoholic use. aOR, adjusted odds ratio, CI, confidence interval.

**Figure 1:**
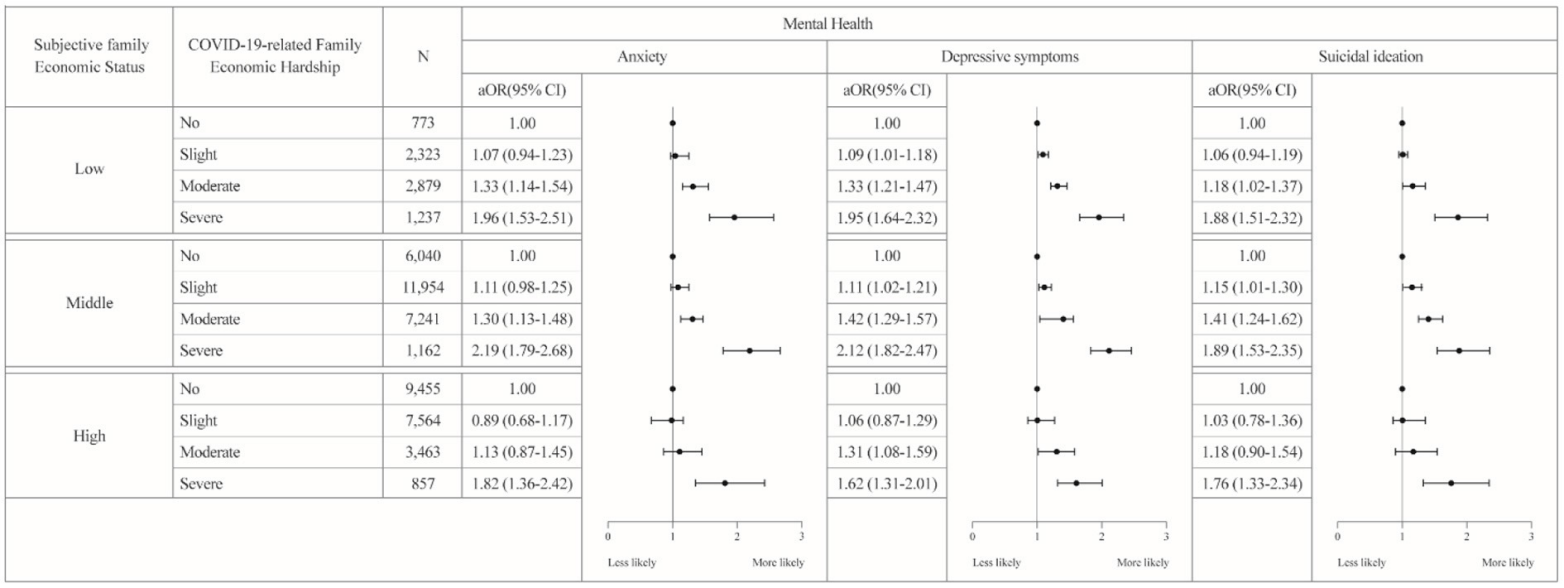
Results of the stratified analyses by subjective family economic status (*N* = 54,948). We performed stratified analyses of the association between COVID-19 related family economic hardship and mental health, stratified by subjective household economic status. We adjusted for sex, grade, residential area, co-residence with parents, subjective academic performance, subjective health status, smoking status, and alcoholic drinking.

Figure 1 presents the results of the stratified analyses. Regarding anxiety, in the group with low subjective family economic status, adolescents who experienced economic hardship were more likely to report anxiety than those who experienced no hardship (experienced moderate hardship aOR = 1.33, 95% CI: 1.14–1.54; experienced severe hardship aOR = 1.96, 95% CI: 1.53–2.51). In the group with mid-level subjective family economic status, the experience of hardship was significantly associated with anxiety (experienced moderate hardship aOR = 1.30, 95% CI: 1.13–1.48; experienced severe hardship aOR = 2.19, 95% CI: 1.79–2.68). In the group with high subjective family economic status, adolescents who experienced hardship were more likely to experience anxiety (experienced severe hardship aOR = 1.82, 95% CI: 1.36–2.42).

Regarding depressive symptoms, in the group with low subjective family economic status, adolescents who experienced hardship were more likely to experience depressive symptoms than adolescents who experienced no hardship (experienced slight hardship aOR = 1.09, 95% CI: 1.01–1.18; experienced moderate hardship aOR = 1.33, 95% CI: 1.21–1.47; experienced severe hardship aOR = 1.95, 95% CI: 1.64–2.32). In the group with mid-level subjective family economic status, the experience of hardship was significantly associated with depressive symptoms (experienced slight hardship aOR = 1.11, 95% CI: 1.02–1.21; experienced moderate hardship aOR = 1.42, 95% CI: 1.29–1.57; experienced severe hardship aOR = 2.12, 95% CI: 1.82–2.47). In the group with high subjective family economic status, adolescents who experienced economic hardship were more likely to have depressive symptoms (experienced moderate hardship aOR = 1.31, 95% CI: 1.08–1.59; experienced severe hardship aOR = 1.62, 95% CI: 1.31–2.01).

Regarding suicidal ideation, in the group with low subjective family economic status, adolescents who experienced economic hardship were more likely to have suicidal ideation than adolescents who experienced no hardship (experienced moderate hardship aOR = 1.18, 95% CI: 1.02–1.37; experienced severe hardship aOR = 1.88, 95% CI: 1.51–2.32). In the group with mid-level subjective family economic status, the experience of a downturn was associated with suicidal ideation (experienced slight hardship aOR = 1.15, 95% CI: 1.01–1.30; experienced moderate hardship aOR = 1.41, 95% CI: 1.24–1.62; experienced severe hardship aOR = 1.89, 95% CI: 1.53–2.35). In the group with high subjective family economic status, adolescents who experienced economic hardship were more likely to experience suicidal ideation (experienced severe hardship aOR = 1.76, 95% CI: 1.33–2.34).

In addition, Table 3 presents the results of the stratified analysis, which was conducted to explore the association between the family economic hardship and mental health, with a special emphasis on sex differences during the COVID-19 pandemic. Compared to the females, the males who had severe economic hardship showed more likely to have anxiety (aOR = 2.13, 95% CI: 1.75–2.60). However, compared to the males more females had depressive symptoms (aOR = 2.15, 95% CI: 1.88–2.45) and suicidal ideation (aOR = 2.04, 95% CI: 1.73–2.4).

**Table 3:** Sex-based association between COVID-19-related family economic hardship and mental health (Male *N* = 28,353; Female *N* = 26,595).

	**Anxiety**		**Depressive symptoms**		**Suicidal ideation**	
**Variables**	**Male**	**Female**	**Male**	**Female**	**Male**	**Female**
	**aOR (95% CI)**	**aOR (95% CI)**	**aOR (95% CI)**	**aOR (95% CI)**	**aOR (95% CI)**	**aOR (95% CI)**
**COVID-19-related family economic hardship**						
No	1.00	1.00	1.00	1.00	1.00	1.00
Slight	1.10 (0.96–1.26)	1.05 (0.94–1.16)	1.03 (0.95–1.12)	1.16 (1.07–1.25)**	1.00 (0.89–1.14)	1.15 (1.04–1.27)**
Moderate	1.22 (1.04–1.44)*	1.32 (1.18–1.48)***	1.28 (1.16–1.41)***	1.47 (1.35–1.60)***	1.18 (1.03–1.34)*	1.36 (1.21–1.53)***
Severe	2.13 (1.75–2.60)***	2.05 (1.72–2.44)***	1.69 (1.46–1.94)***	2.15 (1.88–2.45)***	1.67 (1.40–2.00)***	2.04 (1.73–2.41)***

We adjusted the analyses for sex, grade, residential area, co-residence with parents, subjective academic performance, subjective family economic status, subjective health status, smoking status, alcoholic use.

aOR, odds ratio, CI, confidence interval. ^*^p < 0.05, ^**^p < 0.01, ^***^p < 0.001.

## Discussion

To the best of our knowledge, this is the first nationwide-representative study on COVID-19-related family economic hardship and mental health of adolescents during the COVID-19 pandemic. Our findings highlight that COVID-19 and its resulting economic hardships may contribute to a decrease in mental health among adolescents. As hypothesized in this study, we confirmed that the greater the economic hardship after COVID-19, the more severe the mental health issues (anxiety, depressive symptoms, and suicidal ideation) faced by adolescents. Furthermore, when the association of subjective family economic status with the mental health of adolescents was examined using stratified analysis, we identified that economic hardship caused by the COVID-19 pandemic had a significant impact on the mental health of adolescents belonging to the middle- to low-income economic groups.

Overall, about 11.1% of the participants of our study reported feeling anxiety, 25.2% having depressive symptoms, and 10.9% experiencing suicidal ideation. Our findings align with prior research demonstrating poor mental health during disease outbreaks, as well as with studies that reported high levels of psychological distress in other samples during the COVID-19 pandemic (30, 31) From the total, 70.4% reported experiencing economic hardship. Moreover, over 30% of our sample reported going through moderate and severe economic hardship. These findings showed that adolescents personally experience and perceive economic hardship in their families during the pandemic and highlight that majority of adolescents are experiencing COVID-19-related family economic hardship.

It was found that the greater the COVID-19-related family economic hardships, the worse the anxiety, depressive symptoms, and suicidal ideation. This link coincides with prior research on economic hardship that showed that the mental health of adolescents worsened when the economic circumstances of their parents deteriorated (32–34). Indeed, existing studies have long identified familial poverty as a risk factor for increased mental health problems among adolescents (35–37). In addition, previous studies have reported that a loss of jobs and income for parents negatively affects their children's health (38, 39).

Underemployment or job loss limits economic resources for families, thereby restricting their ability to obtain resources for ensuring consumption, education, food, housing, and a safe environment necessary for the development of adolescents (40). These deficient economic conditions can reduce the psychological resources and parenting quality of parents (41).

Furthermore, earlier studies have shown that mental health is affected by unexpected economic hardship, and that its effect varies between males and females—that is, sex is a crucial factor that must be taken into account (37, 40, 42, 43). Therefore, we analyzed the association between sex-based mental health status and family economic hardship through four subsequent categories. The results of which, demonstrated that, compared to boys, more girls with severe family economic hardship had depressive symptoms and suicidal ideation; whereas, compared to girls, boys with severe economic hardship were more likely to have anxiety. Blackwell et al. (42) reported that the economic hardship during the COVID-19 pandemic led to higher levels of stress, anxiety and depression, particularly among adolescent females. This demonstrates that family economic hardship varies based on sex-based mental health problems. Therefore, specific attention must be paid to sex differences and should be investigated in future research.

With the prolongation of the COVID-19 pandemic, uncertainty surrounding the labor market persists. Furthermore, despite the implementation of income loss compensation plans by many countries worldwide, these efforts focus on economic issues rather than those related to health (44). The potential negative impacts of COVID-19-related family economic hardship on the mental health and well-being of adolescents were evident in our study. Thus, in order to mitigate the negative consequences, health-focused policies and interventions are necessary. It must also be noted that family economic hardship itself, even if only perceived, can also have negative mental health consequences (45, 46). In addition, as poverty is inextricably linked to health, caution is necessary for adolescents in low- to middle-income households who report that they are struggling financially due to COVID-19 (47, 48). Our study emphasizes the need for governments to attend to adolescents who are vulnerable to economic hardship as well as illness during the COVID-19 pandemic so that the discussion of social support to address the economic and health needs of families can take place.

This study has several limitations. First, this study was cross-sectional in design; hence, causal relationships between COVID-19-related family economic hardship and anxiety, depressive symptoms, and suicidal ideation could not be determined because of non-validated measures. However, related literature (49) reporting the association of suicidal ideation and depressive symptoms with household income is most likely based on self-reported responses, which may have influenced the results. Second, all data were collected through self-report questionnaires, and thus, the student-reported information may include some inaccuracy. Third, screening tools like Patient Health Questionnaire (PHQ)-2 and PHQ-9 for depression and Ask Suicide-Screening Questions-4 for suicide could not be used because of the limitations of the data. Finally, our study estimated short-term adolescent mental health responses to shock associated with COVID-19 related family economic hardship; thus, we could not address economy-wide and long-term economic hardships or examine whether the effects on mental health are transitory or persistent. However, even short-term mental health problems can have serious consequences in childhood and adolescence (50, 51). Despite these limitations, our study has several important implications. This study evaluated the association between COVID-19-related family economic hardship and adolescents' mental health during the COVID-19 pandemic using well-defined, nationally representative data in Korea.

## Conclusion

In this study, we investigated the association between COVID-19-related family economic hardship and mental health issues of adolescents. Specifically, we examined anxiety, depressive symptoms, and suicidal ideation. We confirmed that the effect of family economic hardship extends to adolescents, moving beyond the participants of the labor market. These spillover effects on children's mental health suggest that the policy responses to weak economic conditions may have greater effects than anticipated. Therefore, considering the negative impact of mental health disorders (anxiety, depressive symptoms, and suicidal ideation) on daily life and health outcomes, policymakers should consider timely screening and appropriate interventions, such as online psychological counseling tailored for concerns specific to adolescents, to reduce the likelihood of emotional disturbances among adolescents during and after the COVID-19 outbreak.

## Data availability statement

Publicly available datasets were analyzed in this study. This data can be found at: https://www.kdca.go.kr/yhs/.

## Ethics statement

Ethical review and approval was not required for the study on human participants in accordance with the local legislation and institutional requirements. Written informed consent to participate in this study was provided by the participants.

## Author contributions

BK conceptualized the study, cleaned the data, performed the analyses, wrote the initial draft, and led the writing of the final draft. DHK wrote the first draft, and interpreted the results. S-YJ and JS substantially contributed to the design of the analyses, the interpretation of results, and the writing of the final draft. SGL contributed to draft writing and edited writing. THK designed and coordinated the study, supervised statistical analysis, contributed to draft writing and edited writing. All authors have read and approved the final manuscript.

## Conflict of interest

The authors declare that the research was conducted in the absence of any commercial or financial relationships that could be construed as a potential conflict of interest.

## Publisher's note

All claims expressed in this article are solely those of the authors and do not necessarily represent those of their affiliated organizations, or those of the publisher, the editors and the reviewers. Any product that may be evaluated in this article, or claim that may be made by its manufacturer, is not guaranteed or endorsed by the publisher.

## Abbreviations

CI: Confidence intervals
COVID-19: coronavirus disease 2019
KYRBS: Korea Youth Risk Behavior Web-Based Survey
aOR: adjusted Odds Ratios.

## References

[1] Ravens-Sieberer U Kaman A Erhart M Devine J Schlack R Otto C. Impact of the COVID-19 pandemic on quality of life and mental health in children and adolescents in Germany. Eur Child Adolesc Psychiatry. (2021) 25:1–11. 10.2139/ssrn.3721508 33492480 PMC7829493

[2] Crayne MP. The traumatic impact of job loss and job search in the aftermath of COVID-19. Psychol Trauma. (2020) 12:S180–2. 10.1037/tra0000852 32478539

[3] Im J Kim J Choeh JY. COVID-19, social distancing, and risk-averse actions of hospitality and tourism consumers: a case of South Korea. J Destin Mark Manag. (2021) 20:100566. 10.1016/j.jdmm.2021.100566

[4] Gassman-Pines A Ananat EO Fitz-Henley J. COVID-19 and parent-child psychological well-being. Pediatrics. (2020) 146:e2020007294. 10.1542/peds.2020-007294 32764151 PMC7546085

[5] Xie X Xue Q Zhou Y Zhu K Liu Q Zhang J. Mental health status among children in home confinement during the coronavirus disease 2019 outbreak in Hubei Province, China. JAMA Pediatr. (2020) 174:898–900. 10.1001/jamapediatrics.2020.1619 32329784 PMC7182958

[6] Tang S Xiang M Cheung T Xiang YT. Mental health and its correlates among children and adolescents during COVID-19 school closure: the importance of parent-child discussion. J Affect Disord. (2021) 279:353–60. 10.1016/j.jad.2020.10.016 33099049 PMC7550131

[7] Patrick SW Henkhaus LE Zickafoose JS Lovell K Halvorson A Loch S. Well-being of parents and children during the COVID-19 pandemic: a national survey. Pediatrics. (2020) 146:e2020016824. 10.1542/peds.2020-016824 32709738

[8] Li W Wang Z Wang G Ip P Sun X Jiang Y. Socioeconomic inequality in child mental health during the COVID-19 pandemic: first evidence from China. J Affect Disord. (2021) 287:8–14. 10.1016/j.jad.2021.03.009 33761325 PMC9754677

[9] Witteveen D Velthorst E. Economic hardship and mental health complaints during COVID-19. Proc Natl Acad Sci U S A. (2020) 117:27277–84. 10.1073/pnas.2009609117 33046648 PMC7959574

[10] Pierce M Hope H Ford T Hatch S Hotopf M John A. Mental health before and during the COVID-19 pandemic: a longitudinal probability sample survey of the UK population. Lancet Psychiatry. (2020) 7:883–92. 10.1016/S2215-0366(20)30308-4 32707037 PMC7373389

[11] Prime H Wade M Browne DT. Risk and resilience in family well-being during the COVID-19 pandemic. Am Psychol. (2020) 75:631–43. 10.1037/amp0000660 32437181

[12] Fröjd S Marttunen M Pelkonen M von der Pahlen B Kaltiala-Heino R. Perceived financial difficulties and maladjustment outcomes in adolescence. Eur J Public Health. (2006) 16:542–8. 10.1093/eurpub/ckl012 16641162

[13] Shek DT. Economic stress, psychological well-being and problem behavior in Chinese adolescents with economic disadvantage. J Youth Adolesc. (2003) 32:259–66. 10.1023/A:1023080826557

[14] Karpman M Zuckerman S Gonzalez D Kenney GM. The COVID-19 Pandemic is Straining Families' Abilities to Afford Basic Needs. Washington, DC: Urban Institute (2020). p. 500.

[15] UNICEF UNDP Prospera the SMERU Research. Analysis of the Social and Economic Impacts of COVID-19 on Households and Strategic Policy Recommendations for Indonesia. (2021). Available online at: https://www.unicef.org/indonesia/media/9501/file/Analysis%20of%20the%20Social%20and%20Economic%20Impacts%20of%20COVID-19%20on%20Household%20and%20Strategic%20Policy%20Recommendations%20for%20Indonesia.pdf (accessed June 10, 2022)

[16] Wickrama KAS Surjadi FF Lorenz FO Conger RD Walker C. Family economic hardship and progression of poor mental health in middle-aged husbands and wives. Fam Relat. (2012) 61:297–312. 10.1111/j.1741-3729.2011.00697.x 22577243 PMC3346274

[17] Lee TK Wickrama KA Simons LG. Chronic family economic hardship, family processes and progression of mental and physical health symptoms in adolescence. J Youth Adolesc. (2013) 42:821–36. 10.1007/s10964-012-9808-1 22927008

[18] Sampasa-Kanyinga H Hamilton HA. Social networking sites and mental health problems in adolescents: the mediating role of cyberbullying victimization. Eur Psychiatry. (2015) 30:1021–7. 10.1016/j.eurpsy.2015.09.011 26512450

[19] Li G Mei J You J Miao J Song X Sun W. Sociodemographic characteristics associated with adolescent depression in urban and rural areas of Hubei province: a cross-sectional analysis. BMC Psychiatry. (2019) 19:386. 10.1186/s12888-019-2380-4 31805901 PMC6896285

[20] Assing-Murray E. Lebrun-Harris L. Associations between parent-reported family economic hardship and mental health conditions in US children. J Child Poverty. (2020) 26:191–214. 10.1080/10796126.2020.1764188

[21] Kato TA Sartorius N Shinfuku N. Forced social isolation due to COVID-19 and consequent mental health problems: lessons from hikikomori. Psychiatry Clin Neurosci. (2020) 74:506–7. 10.1111/pcn.13112 32654336 PMC7404367

[22] Creswell C Shum A Pearcey S Skripkauskaite S Patalay P Waite P. Young people's mental health during the COVID-19 pandemic. Lancet Child Adolesc Health. (2021) 5:535–7. 10.1016/S2352-4642(21)00177-2 34174991 PMC9765398

[23] Kim Y Choi S Chun C Park S Khang YH Oh K. Data resource profile: the Korea Youth Risk Behavior Web-based Survey (KYRBS). Int J Epidemiol. (2016) 45:1076–1076e. 10.1093/ije/dyw070 27380796

[24] World Medical Association. World Medical Association Declaration of Helsinki: ethical principles for medical research involving human subjects. JAMA. (2013) 310:2191–4. 10.1001/jama.2013.281053 24141714

[25] Spitzer RL Kroenke K Williams JB Löwe B. A brief measure for assessing generalized anxiety disorder: the GAD-7. Arch Intern Med. (2006) 166:1092–7. 10.1001/archinte.166.10.1092 16717171

[26] Löwe B Decker O Müller S Brähler E Schellberg D Herzog W. Validation and standardization of the Generalized Anxiety Disorder Screener (GAD-7) in the general population. Med Care. (2008) 46:266–74. 10.1097/MLR.0b013e318160d093 18388841

[27] Mossman SA Luft MJ Schroeder HK Varney ST Fleck DE Barzman DH. The Generalized Anxiety Disorder 7-item scale in adolescents with generalized anxiety disorder: signal detection and validation. Ann Clin Psychiatry. (2017) 29:227–34. Available online at: https://www.aacp.com/article/buy_now/?id=499 29069107 PMC5765270

[28] Nunnally JC. Psychometric Theory. 2nd Ed. New York, NY: McGraw-Hill (1978).

[29] Mack J Lansley S. Poor Britain. London: G Allen & Unwin (1985).

[30] Kock M Kuppens P Van der Gucht K Raes F. Mindfulness may buffer psychological distress in adolescents during the COVID-19 pandemic: the differential role of mindfulness facets. Psychol Belg. (2021) 61:356–76. 10.5334/pb.1093 34900325 PMC8622000

[31] Sun S Goldberg SB Lin D Qiao S Operario D. Psychiatric symptoms, risk, and protective factors among university students in quarantine during the COVID-19 pandemic in China. Global Health. (2021) 17:1–14. 10.1186/s12992-021-00663-x 33494769 PMC7829620

[32] Ananat EO Gassman-Pines A Francis DV Gibson-Davis CM. Linking job loss, inequality, mental health, and education. Science. (2017) 356:1127–8. 10.1126/science.aam5347 28619903

[33] Catalano R Goldman-Mellor S Saxton K Margerison-Zilko C Subbraman M LeWinn K. The health effects of economic decline. Annu Rev Public Health. (2011) 32:431–50. 10.1146/annurev-publhealth-031210-101146 21054175 PMC3855327

[34] Gassman-Pines A Ananat EO Gibson-Davis CM. Effects of statewide job losses on adolescent suicide-related behaviors. Am J Public Health. (2014) 104:1964–70. 10.2105/AJPH.2014.302081 25122027 PMC4167092

[35] Dearing E. Psychological costs of growing up poor. Ann N Y Acad Sci. (2008) 1136:324–32. 10.1196/annals.1425.006 17954672

[36] Letourneau NL Duffett-Leger L Levac L Watson B Young-Mosrris C. Socioeconomic status and child development: a meta-analysis. J Emot Behav Disord. (2013) 21:211–24. 10.1177/1063426611421007

[37] Reiss F. Socioeconomic inequalities and mental health problems in children and adolescents: a systematic review. Soc Sci Med. (2013) 90:24–31. 10.1016/j.socscimed.2013.04.026 23746605

[38] Kalil A. Joblessness, family relations, and children's development. Fam Matters. (2009) (83):15–22. Available online at: https://aifs.gov.au/research/family-matters/no-83/joblessness-family-relations-and-childrens-development

[39] McLoyd VC. Socialization and development in a changing economy: the effects of paternal job and income loss on children. Am Psychol. (1989) 44:293. 10.1037/0003-066X.44.2.293

[40] Yoshikawa H Aber JL Beardslee WR. The effects of poverty on the mental, emotional, and behavioral health of children and youth: implications for prevention. Am Psychol. (2012) 67:272–84. 10.1037/a0028015 22583341

[41] Yoshikawa H Weisner TS Lowe ED. Making it Work: Low-Wage Employment, Family Life, and Child Development. Manhattan, NY: Russell Sage Foundation (2006).

[42] Blackwell CK Mansolf M Sherlock P Ganiban J Hofheimer JA Barone CJ. Youth well-being during the COVID-19 pandemic. Pediatrics. (2022) 149:e2021054754. 10.1542/peds.2021-054754 35301542 PMC9169239

[43] Wadsworth ME Raviv T Santiago CD Etter EM. Testing the adaptation to poverty-related stress model: predicting psychopathology symptoms in families facing economic hardship. J Clin Child Adolesc Psychol. (2011) 40:646–57. 10.1080/15374416.2011.581622 21722035

[44] Gray BJ Kyle RG Song J Davies AR. Characteristics of those most vulnerable to employment changes during the COVID-19 pandemic: a nationally representative cross-sectional study in Wales. J Epidemiol Commun Health. (2022) 76:8–15. 10.1136/jech-2020-216030 34193569 PMC8249173

[45] Kim TJ von dem Knesebeck O. Is an insecure job better for health than having no job at all? A systematic review of studies investigating the health-related risks of both job insecurity and unemployment. BMC Public Health. (2015) 15:985. 10.1186/s12889-015-2313-1 26419739 PMC4589035

[46] Kim TJ von dem Knesebeck O. Perceived job insecurity, unemployment and depressive symptoms: a systematic review and meta-analysis of prospective observational studies. Int Arch Occup Environ Health. (2016) 89:561–73. 10.1007/s00420-015-1107-1 26715495

[47] Marmot M. Health equity in England: the marmot review 10 years on. BMJ. (2020) 368:m693. 10.1136/bmj.m693 32094110

[48] Lai ET Wickham S Law C Whitehead M Barr B Taylor-Robinson D. Poverty dynamics and health in late childhood in the UK: evidence from the millennium cohort study. Arch Dis Child. (2019) 104:1049–55. 10.1136/archdischild-2018-316702 31186294 PMC6837248

[49] Han JM Song H. Effect of subjective economic status during the Covid-19 pandemic on depressive symptoms and suicidal ideation among South Korean adolescents. Psychol Res Behav Manag. (2021) 14:2035–43. 10.2147/PRBM.S326660 34934369 PMC8684414

[50] Busch SH Golberstein E Meara E. The FDA and ABCs: unintended consequences of antidepressant warnings on human capital. J Hum Resour. (2014) 49:540–71. 10.1353/jhr.2014.0016 25284886 PMC4181847

[51] Currie J Stabile M. Mental health in childhood and human capital. In: Gruber J editor. The Problems of Disadvantaged Youth: An Economic Perspective. Chicago, IL: University of Chicago Press (2009). p. 115–148.

